# Angiomatoid fibrous histiocytoma with EWSR1-CREB1 gene fusion occurs in lungs and ribs with systemic multiple metastases: a case report and review of the literature

**DOI:** 10.3389/fonc.2024.1420597

**Published:** 2025-01-08

**Authors:** Dongmei Feng, Ying Li, Zhengjin Li, Yun Pan, Yixuan Gao, Jinyan Cha, Chunmei Zhang

**Affiliations:** ^1^ Department of Pathology, The First Affiliated Hospital of Dali University, Dali, Yunnan, China; ^2^ Department of Pathology, People’s Hospital of Xiangyun County, Xiangyun, Yunnan, China

**Keywords:** angiomatoid fibrous histiocytoma, bone, EWSR1-CREB1, lung, metastasis

## Abstract

Angiomatoid fibrous histiocytoma (AFH) is a rare soft tissue tumor with intermediate malignant potential, and it rarely metastasizes. We encountered a unique AFH case where, the tumor was discovered initially in unusual locations—the left lung and the left 4th rib. Combined histological features with FISH and NGS analysis, the diagnosis of AFH was supported, however, it is difficult to determine which of these two is the primary lesion. Eight months after the initial surgery, multiple systemic metastases were detected, eventually leading to the patient’s death 18 months later due to widespread metastasis. Our case signifies the first reported occurrence of systemic metastasis in either bone-originating or pulmonary-originating AFH, and it is the initial instance of mortality resulting from multifocal metastasis originating from an atypical site.

## 1 Introduction

Angiomatoid fibrous histiocytoma (AFH), initially described by Enzinger in 1979, was classified by the World Health Organization in 2020 as a “differentiation uncertain tumor” with moderate biological potential and unclear differentiation, accounting for 0.3% of soft tissue tumors (1). While the prognosis for most AFH patients is favorable, approximately 15% experience local recurrence, and 1-5% develop distant metastases (2). AFH predominantly occurs in the superficial extremities of children and young adults, with rare cases reported in non-trunk soft tissue locations such as the lungs, mediastinum, bones, reproductive system, oral cavity, adrenal glands, skull, breasts, and spinal canal. Clinical symptoms are related to the site of onset (3). We present a rare case of AFH, initially occurring in uncommon sites—the lungs and ribs—followed by widespread metastasis, ultimately leading to the patient’s death due to disease progression.

## 2 Case report

**Figure 1:**
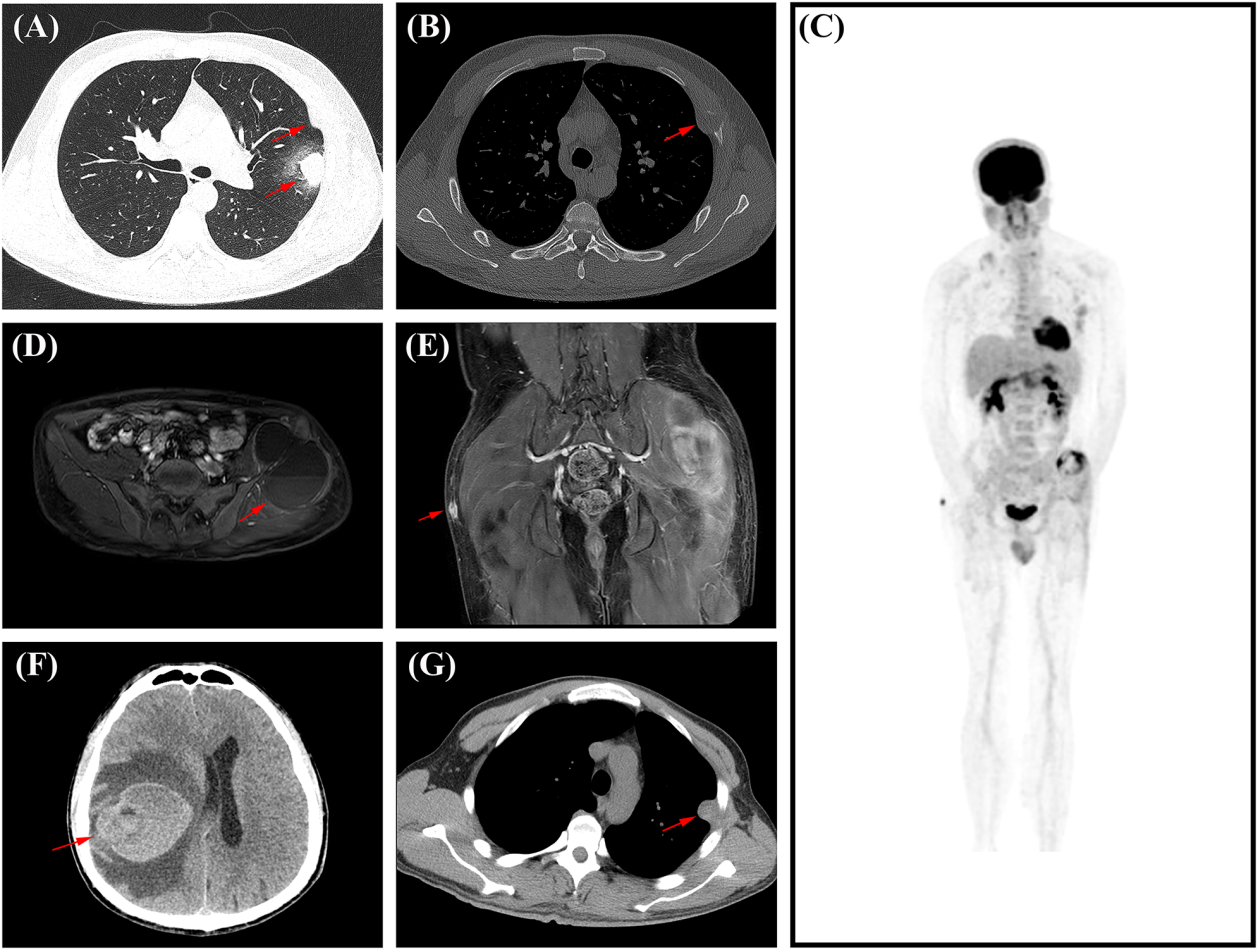
The imaging data of this patient’s disease progression. **(A)** In August 2022, chest CT showed a 2.8cm×2.1cm mass in the upper lobe of the left lung. **(B)** In August 2022, on chest CT, the red arrow refers to the left fourth rib lesion. **(C)** In May 2023, PET/CT showed multiple metastases throughout the body. **(D)** In May 2023, Axial T1 MRI contrast of the pelvis shows metastases in and around the left iliac bone, measuring 9.6 cm×6.4 cm, with a visible fluid-fluid level within it (arrow). **(E)** In May 2023, coronal T1 MRI enhancement showed a subcutaneous nodule on the right buttock, approximately 1.1 cm×1.0cm in size (arrow). **(F)** In October 2023, brain CT showed large patches of hyperdense opacities in the right basal ganglia and frontoparial lobe, with the maximum level ranging from about 6.2cm×5.0cm (arrow). **(G)** In October 2023, chest CT showed a soft tissue nodular opacity at the base of the left pleura, about 1.6 cm×2.6cm in size (arrow).

A 34-year-old male presented with intermittent left chest pain, accompanied by cough and sputum on July 10, 2022. Chest Computed tomography (CT) scan revealed a nodular high-density shadow of 2.5cm×1.9cm in the lingual segment of the left upper lobe, exhibiting clear borders and surrounded by ground-glass opacities. Localized bone expansion and cortical interruption with soft tissue density filling in the left 4th rib were observed, which were mistakenly considered by the radiologist to be caused by inflammation. The patient was readmitted to the hospital on August 7, 2022, for no improvement. A subsequent CT scan showed an enlargement of the pulmonary lesion to 2.8cm×2.1cm, with no significant changes in the left 4th rib lesion, raising suspicions of a tumorous condition (**Figures 1A, B**). Preoperative biopsies of the pulmonary lingual segment nodule and the left 4th rib lesion revealed consistent histological features, sparking suspicion of malignancy. However, a specific diagnosis could not be confirmed based on the histopathological findings and immunohistochemical analysis of the biopsy specimen.

The patient underwent video-assisted thoracoscopic surgery (VATS) on September 6, 2022. The procedure included wedge resection of the left upper lung lobe and excision of the lesion in the left 4th rib. VATS revealed the lung tumor located in the posterior segment of the left upper lung apex, measuring 2.0×2.0×1.5cm, with no observed pleural depression. The lesion in the left 4th rib exhibited unclear boundaries, a firm texture, and measured 2.0×2.0×1.0cm. No residual tumor was observed at the margins of the lung and rib lesions. However, the pathological examination still failed to yield a specific diagnosis.

Eight months post-surgery, the patient sought medical attention at another hospital due to left iliac region pain. The Magnetic resonance imaging (MRI) on May 21, 2023, revealed bone destruction in the left iliac bone, accompanied by a surrounding mixed solid and cystic mass measuring approximately 9.0cm × 6.4cm, exhibiting a fluid-fluid level (**Figure 1D**). Additionally, a subcutaneous nodule measuring 1.1cm×1.0cm was identified in the right buttock, raising concerns about metastases from lung or rib tumors (**Figure 1E**). A 18-fluorodeoxyglucose (FDG) positron emission tomography with CT (PET/CT) revealed increased metabolic activity in multiple regional lymph nodes in the chest, localized regions of the left 4th-7th ribs and adjacent chest wall, and increased metabolism associated with localized bone destruction in the right 11th posterior rib. Soft tissue mass formation and invasion into the left gluteus medius and lateral fascia of the upper thigh were noted in the left iliac bone region, suggesting metastasis. A subcutaneous nodule in the right buttock showed increased metabolic activity, again indicating potential metastasis from lung or rib tumors (**Figure 1C**). To obtain a definitive diagnosis, the patient underwent another surgical procedure to remove the left iliac bone and surrounding lesions. External specialist consultation revealed a diagnosis of AFH. In October 2023, the patient experienced symptoms such as dizziness and headaches, leading to another hospitalization for treatment. A CT examination revealed the presence of metastatic lesions in the brain and pleura (**Figures 1F, G**). In March 2024, the patient died from the progression of AFH, 18 months following the initial surgery.

**Figure 2:**
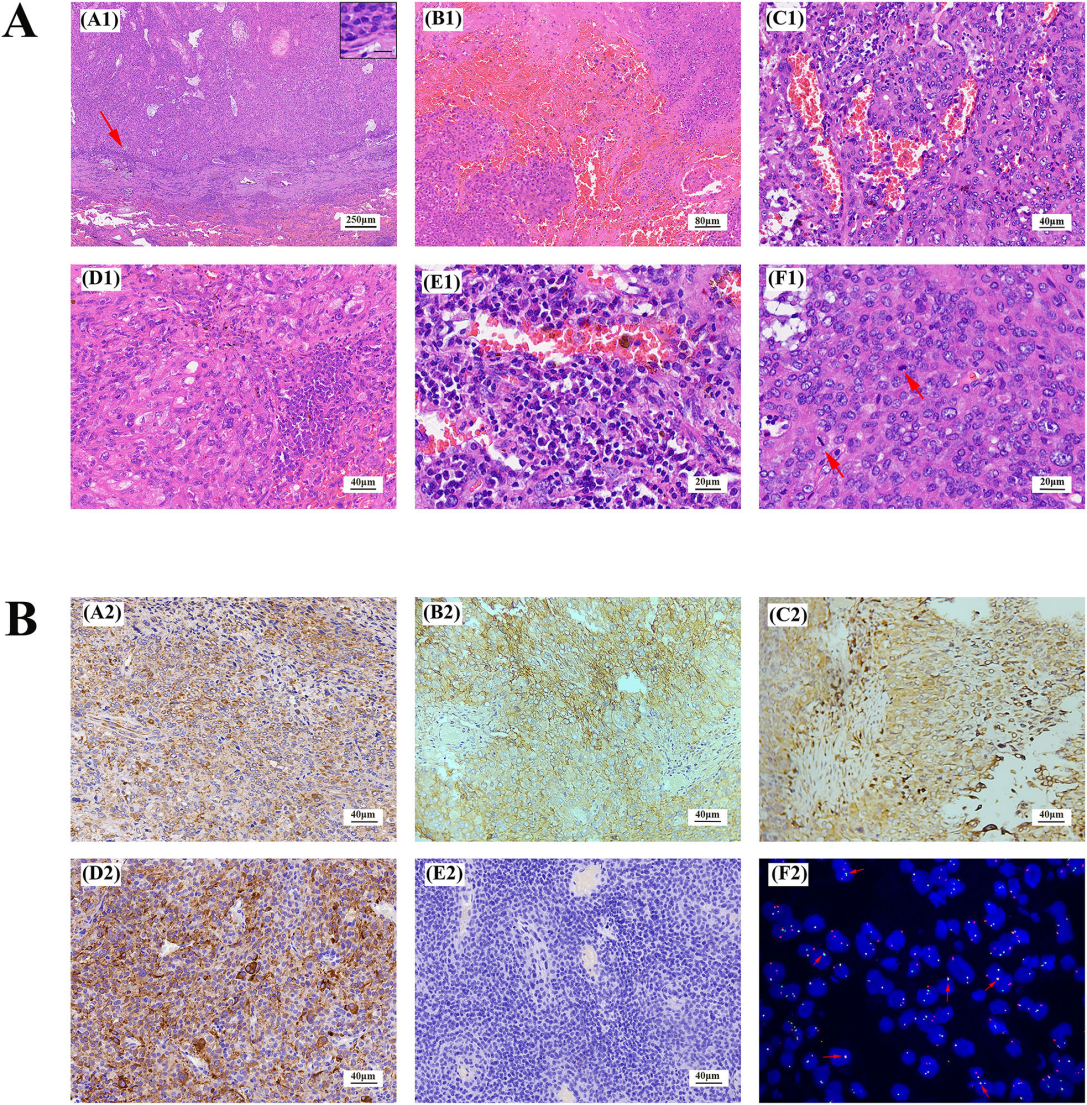
Lung AFH stained with hematoxylin and eosin **(A)**. (A1) There is a distinct fibrous pseudocapsule and pericapsular sleeve of lymphoplasma cells around the tumor nodule (×40). Inset: Lymphoplasma cells densely distributed around the fibrosham capsule (×400), the scale represents 20μm. (B1) A pseudohematous hemorrhagic sac cavity may be seen within the tumor (×100). Slit hemorrhagic areas with hemosiderin deposition and lymphocyte plasmacytic infiltration may be seen within the tumor (×200). (D1) Tumors are mainly composed of fat spindle cells and ovoid cells (×200). (E1) There is more plasma cell infiltration within the tumor (×400). (F1) A mitotic image is visible within the tumor, indicated by arrows (×400). **(B)** Immunohistochemical staining and fluorescence *in situ* hybridization of AFH. Tumor cells were positive for Vimentin, CD99, CD68, Desmin (A2-D2), and negative for S-100 (E2). (F2) Fluorescence *in situ* hybridization analysis revealed fusion of EWSR1 and CREB1. The red fluorescence signal is the CREB1 (2q33) probe, and the green fluorescence signal is the EWSR1 (22q12) probe. The arrow indicates the yellow fluorescence signal, which indicates fusion of EWSR1 and CREB1. (magnification, ×200 for all, tumor cells with brownish−yellow staining are immunohistochemically positive).

Histologically, the left upper lobe tumor exhibited consistent tissue morphology with the rib lesion and left iliac bone lesion. The tumor had clear boundaries, and the surrounding area showed chronic inflammatory cell infiltration, predominantly lymphocytes and plasma cells, forming a sleeve-like structure and enveloped by a thick fibrous pseudo-capsule. The tumor mainly consisted of spindle-shaped, plump spindle-shaped, and oval-shaped cells, arranged in bundles or irregular patterns, exhibiting mild atypia without nuclear pleomorphism or deep staining. Clearly visible nucleoli and 0-2 mitotic figures/10 HPF were observed, with no necrosis. Focal or scattered plasma cell infiltration and hemosiderin deposition were seen in the tumor stroma. Multiple bleeding areas were observed within the tumor cell nests, presenting as pseudo-vascular cystic or cleft-like structures without endothelial cell lining (**Figure 2A**). Immunohistochemistry revealed positive staining for Vimentin, CD68, CD99, EMA, and Desmin (**Figures 2B A2-D2**), weak positive staining (10%) for P53, negative staining for CD21, CD34, S-100 (**Figures 2B E2**), Myoglobin, CK-P, P63, SMA, calponin, and CD21. Fluorescence *in situ* hybridization (FISH) analysis showed rearrangement of the EWSR1 gene with CREB1, confirming the presence of the EWSR1-CREB1 fusion gene (**Figures 2B F2**). RNA level next-generation sequencing (NGS) directly confirmed the fusion of the 7th exon of the EWSR1 gene with the 7th exon of the CREB1 gene. These findings supported the definitive diagnosis of angiomatoid fibrous histiocytoma in the lungs, ribs, and left iliac bone.

## 3 Discussion

The etiology of AFH remains enigmatic. Multipotent mesenchymal stem cells are considered a potential source, while there are viewpoints suggesting that AFH may emerge as a secondary malignancy in various cancer patients, including those with HIV (4).

AFH presents with extensive morphological characteristics, comprising four primary histological features (5). Firstly, the tumor consists of irregularly distributed polygonal, spindle, oval, and round cells with histiocytic or myoid features. Secondly, the tumor nests exhibit multifocal hemorrhagic cystic spaces devoid of endothelial cell lining, resembling pseudo-vascular cavities or slit-like clefts. The third hallmark is the presence of a thick and incomplete fibrous pseudo-capsule. Lastly, the tumor is densely infiltrated by lymphoplasmacytic cells or cystic envelopes, potentially including the formation of germinal centers (5).

While many reported cases lack all four major histological features simultaneously, the first feature remains consistently invariant (6). Our case encompasses all four primary histological features. Although immunohistochemical staining may provide some support for AFH diagnosis, specific markers are lacking. Approximately half of the cases express desmin, while the expression of epithelial membrane antigen, CD99, and CD68 varies, reported in approximately 40% to 50% of cases (6).

Due to its rarity, extensive histomorphological characteristics, and lack of a specific immunological spectrum, diagnosing AFH becomes extremely challenging when the lesion occurs in unusual locations and exhibits rare biological behaviors. However, molecular studies are particularly helpful for diagnosing AFH with unusual locations and rare biological behaviors such as metastasis. The majority of AFH cases display unique chromosomal translocations, with three common translocations: EWSR1/CREB1 (most frequent), EWSR1/ATF1, and FUS/ATF1 gene fusions (7). In our case, Fluorescence *in situ* hybridization (FISH) testing revealed the EWSR1/CREB1 gene fusion. RNA level next-generation sequencing (NGS) confirmed the fusion of exon 7 of the EWSR1 gene with exon 7 of the CREB1 gene, supporting the main histological features of AFH, along with immunohistochemical support for vimentin, CD99, and CD68.

In our case, the lesion in the left iliac bone and surrounding areas exhibited fluid-fluid levels (FFL) on imaging. Histologically, these lesions were characterized by blood-filled cystic spaces lined with tumor cells rather than endothelial cells, a characteristic feature of AFH. This histological feature was observed in all surgically excised lesions in the patient. The pathophysiological mechanism underlying the formation of FFL is not yet fully understood (8). Besides AFH, other cystic lesions with liquefactive necrosis, hemorrhagic tumors, abscesses, chronic hematomas, epidermal cysts, and complex cysts can also present with FFL (8, 9). FFL are not specific to any particular lesion and can occur in benign tumors, malignant tumors, and non-neoplastic entities (8). In soft tissue and bone tumors, FFL are commonly seen in synovial sarcoma(SS), aneurysmal bone cyst(ABC), telangiectatic osteosarcoma(TO), and Ewing sarcoma(ES), all of which need to be differentiated from each other (8). SS primarily affects individuals aged 15 to 40 and is most common in the extremities, especially the lower limbs (10). SS often presents as a multilocular mass with internal septations or cyst formation, exhibiting FFL on MR imaging. Histologically, there are various subtypes, with cyst formation frequently observed in the monophasic variant. The cysts have smooth walls and contain mucoid fluid or blood (10, 11). ABC is most common in patients with immature skeletons, particularly within the first 20 years of life (12). It widely affects the skeleton, with the cranial bones, vertebrae, and metaphysis of long tubular bones being more commonly involved (12). Histologically, ABC presents as multiloculated cystic lesions with cyst walls collapsing against a background of hemorrhage (12). The cysts typically lack any lining, but flattened endothelial-like cells may be seen (12). TO typically affects individuals aged 15 to 20 and most commonly occurs in the metaphyses of long bones such as the distal femur, proximal tibia, and proximal humerus (13). Histologically similar to ABC, TO lesions are primarily composed of hemorrhage and necrotic debris, with blood pools lacking an endothelial layer (13, 14). Septa containing atypical stromal cells of varying sizes can be found within these blood lakes (13, 14). ES predominantly affects individuals aged 10 to 19, with most cases arising in the skeleton (15). Skeletal ES most commonly affects the diaphyses or metaphyses of long bones (15). Pathologically, areas of hemorrhage and necrosis are commonly observed, leading to soft or partially liquefied regions resembling purulent exudate (15). Although these tumors exhibit FFL on imaging, their corresponding histological features do not necessarily include blood-filled pseudovascular cystic spaces.

Due to the diverse histological characteristics of AFH, its differential diagnosis encompasses a wide range of conditions. The first differential diagnosis to consider is aneurysmal fibrous histiocytoma, which retains the typical structural characteristics of dermatofibroma, such as epidermal hyperplasia and peripheral collagen bundles (16). Blood-filled pseudocysts, lacking an endothelial lining and surrounded by storiform-arranged spindle cells, can be observed (17). This tumor does not exhibit the genetic alterations seen in AFH (17). The second differential diagnosis is inflammatory myofibroblastic tumor (IMT). IMT and AFH exhibit spindle tumor cells with lymphocyte and plasma cell infiltration (7). However, IMT lacks other characteristic morphological features and molecular genetic alterations that are characteristic of AFH (7). In our case, one of the two lesions identified during the patient’s initial presentation was located in the lung. Although it was unclear if it was a primary site, differentiation from primary pulmonary myxoid sarcoma (PPMS) was necessary. Primary pulmonary AFH (PPAFH) and PPMS show significant overlap in clinical, pathological, and molecular characteristics (7). PPMS is predominantly a myxoid tumor with a myxoid stroma comprising up to 30% of the tumor, whereas in PPAFH, it is only focal (7). PPAFH’s distinctive histological features, such as a fibrous pseudocapsule, lymphoplasmacytic infiltrate, and fibrosclerosis, are more common compared to PPMS, which lacks a peritumoral lymphoplasmacytic cuff. EWSR1 rearrangement is found in 100% of PPAFH cases and in 79% of PPMS cases, with EWSR1-ATF1 fusion present in 37.5% of PPAFH but rarely in PPMS (7). Finally, metastasis of a fibrohistiocytic tumor to a lymph node should be considered in the differential diagnosis. AFH’s typical histological features include a fibrous pseudocapsule and dense lymphoplasmacytic infiltration or pericystic arrangement around the tumor, sometimes forming germinal centers (18). These features can lead to confusion when fibrohistiocytic tumors metastasize to lymph nodes (18). Imaging studies or a detailed patient history may help identify the primary site, and histological examination for the presence or absence of subcapsular and medullary sinuses, along with further molecular testing, can aid in differentiating between AFH and metastasis of a fibrohistiocytic tumor to the lymph node (6).

Primary pulmonary AFH is exceedingly rare, first reported in 2009 (19). According to current English literature records, only 16 cases are documented (**Table 1**) (4, 7, 19–27). Among these cases, only one had localized lymph node metastasis. The case reported by G. Bermudo et al. did not specify whether the subcutaneous lesion was a metastasis (26). Documented cases of AFH originating in bones are only 10, with one case showing inguinal lymph node metastasis (**Table 1**) (20, 28–34). No cases of AFH originating in the ribs have been reported. Our case is the first instance of widespread systemic metastasis, regardless of whether originating in bone or lungs. It is also the first reported case of mortality resulting from multifocal metastasis originating from an atypical site.

**Table 1:** Literature describing the clinical and pathology features of AFH in the primary lungs and bones.

	Sex/Age (years)	Location	Treatment	Size	Fibrous capsule	Lymphopl-asmatic infiltrate	Pseudoh-emangio- matous lumen	Molecular genetics	Outcome	References
1	M/46	RLL	Lobectomy	25 mm	Partially surrounded	Present	No	EWSR1-ATF1	Well at 24ms after excision	Ren et al. (19)
2	F/60	LUL	Excision	15 mm	Partially surrounded	Present	NA	EWSR1-CREB1	Well at 24ms after excision	Chen et al. (20)
3	M/43	Lung	Excision	24 mm	Partially surrounded	Present	NA	NA	NA	Chen et al. (20)
4	M/64	Endotracheal(LLL)	Lobectomy	15 mm	Partially surrounded	Present	No	EWSR1-CREB1	NA	Thway et al. (21)
5	M/61	Endotracheal (RMB)	Sleeve resection	15 mm	Partially surrounded	No	No	EWSR1-ATF1	NA	Thway et al. (21)
6	M/27	Endotracheal	Sleeve resection	31 mm	NA	No	Present	EWSR1 rearranged	NA	Chen et al. (22)
7	F/70	RUL	Wedge resection+ excision of the lymph node	13 mm	Partially surrounded	Present	No	EWSR1 rearranged	NA	Tay et al. (23)
8	NA/NA	NA	NA	NA	NA	NA	NA	EWSR1 rearranged	NA	Cheah et al. (24)
9	F/22	Endotracheal	HBSR	15 mm	Present	Present	Present	EWSR1-CREB1	Well at 36ms after excision	Bouma et al. (25)
10	M/50	LLL	Wedge resection	20 mm	Present	Present	No	EWSR1-CREB1	Well at 22ms after excision	Wang et al. (7)
11	F/33	RML	Lobectomy	80 mm	Present	Present	Present	EWSR1-CREB1	Well at 17ms after excision	Wang et al. (7)
12	F/55	LUL	Lobectomy	15 mm	Present	Present	No	EWSR1-CREB1	Well at 13ms after excision	Wang et al. (7)
13	M/35	RLL	Lobectomy	15 mm	Present	Present	No	EWSR1-CREB1	Well at 30ms after excision	Wang et al. (7)
14	M/29	Endotracheal (LLL)	Lobectomy+ excision of the lymph node	35 mm	Present	Present	Present	EWSR1 rearranged	NA	Çetin et al. (4)
15	M/25	Upper lobes of both lungs	Excision	NA	NA	NA	Present	NA	NA	Bermudo et al. (26)
16	F/40	LLL	Excision	NA	NA	NA	NA	EWSR1-CREB1	NA	Vargas et al. (27)
17	34/F	Left tibia	Excision	N/A	N/A	N/A	N/A	N/A	Well at 60ms after excision ^a^	Enzinger et al. (28)
18	M/11	Right proximal humerus	Excision	50 mm	NA	NA	Present	EWSR1-ATF1	Well at 16ms after excision	Mangham et al. (29)
19	M/5	Left ischium	Treated by curettage and Excision	NA	Present	Present	Present	EWSR1 rearranged	Recurrence at 12ms	Petrey et al. (30)
20	F/7	Right mandible	NA	NA	NA	NA	NA	NA	NA	Chen et al. (20)
21	M/8	Right proximal humerus	Treated by curettage and Excision	NA	NA	NA	NA	NA	Recurrence at 3ms and 9ms	Chen et al. (20)
22	M/47	Right proximal ulnar shaft	Treated by curettage and Excision	NA	NA	NA	NA	NA	Recurrence at 84ms	Chen et al. (20)
23	M/10	Left parietal bone	Excision	30 mm	Present	Present	Present	NA	Well at 12ms after excision	Zheng et al. (31)
24	M/54	Right shoulder blade	Excision	45 mm	NA	Present	Present	EWSR1-CREB1	Well at 60ms after excision	Kobayashi et al. (32)
25	F/17	Right temporal bone	Excision	18 mm	Present	Present	No	EWSR1-ATF1	Well at 6ms after excision	Gillon et al. (33)
26	M/42	Right mandible	Excision	39 mm	Present	No	Present	EWSR1-ATF1	Well at 17ms after excision	Hu et al. (34)

F, female; HBSR, hybrid bronchoscopic and surgical resection; LLL & LUL & RMB & RML & RUL (L, left; R, right; LL, lower lobe; MB, main bronchus; ML, middle lobe; UL, upper lobe); M, male; NA, not available data; No, no present.

a The tumor in the left tibial region recurred 3 months after resection and metastasized to the inguinal lymph node one year later. The patient did not recur or metastasize 5 years after surgical resection of recurrent and metastatic lesions.

The primary treatment for AFH is extensive surgical excision. The local recurrence rate is approximately 15%, associated with incomplete initial excision (35). The metastasis rate of AFH is less than 5%, most commonly involving regional lymph nodes and sometimes affecting the lungs, liver, and brain (6). Ossama M. Maher et al. conducted a retrospective analysis of reported cases of AFH with metastasis from 1979 to 2015, encompassing a total of 17 cases, including their own documented case (36). None of these metastatic AFH cases originated in the lungs, and only one case originated in the bones, involving inguinal lymph node metastasis from a primary lesion in the tibia has been counted in **Table 1** (36). These cases exhibited significant variation in the timing of metastasis, ranging from 5 months to 16 years after the primary tumor excision, resulting in extremely rare deaths due to distant metastasis. Overall, the prognosis of AFH is not poor, but due to the potential for recurrence and metastasis, long-term follow-up is recommended.

## Data Availability

The original contributions presented in the study are included in the article/**Supplementary Material**. Further inquiries can be directed to the corresponding author.
