# Supplementary material for: Angiomatoid fibrous histiocytoma with EWSR1-CREB1 gene fusion occurs in lungs and ribs with systemic multiple metastases: a case report and review of the literature
Source: Front Oncol. 2025 Jan 8;14:1420597. doi: 10.3389/fonc.2024.1420597 (PMC11750646; doi:10.3389/fonc.2024.1420597)
Supplement: Supplementary file 2 [file DataSheet2.pdf]

Table 2 Immunohistochemical labeling of biopsy specimens

Positive staining（+）	Negative staining（-）
Ki-67(+5%), ALK(±), EMA(+), CD117(±), Vimentin(+)	CK-Pan(-), LCA/CD45(-), CD38(-), D2-40(-), CD3(-), CD20(-), CD138(-) P63(-), HMB-45(-), Melan-A(-), CD79(-), SALL4(-), S-100(-), CD34(-), TTF-1(-), Desmin(-), SMA(-), NapsinA(-)

Table 3 Immunohistochemical labeling of surgical specimens of lung tumors

Positive staining（+）	Negative staining（-）
Ki-67(+10%), ALK(±), EMA(+), CD117(±), Vimentin(+), Desmin(+), TFE3(+), P53(weak positive + 10%), β-Catenin(±), CD68(+), CD99(±)	CD21(-), LCA/CD45(-), TTF-1(-), AE1/AE3(-), CD10(-), PR(-), Myoglobin(-), CD34(-), calponin(-), D2-40(-),WT-1(-), CD5(-), S-100(-) CD56(-), CgA(-), Syn(-), SALL4(-), calretinin(-)
